# Influence of Stress Factors Related to Cheese-Making Process and to STEC Detection Procedure on the Induction of Stx Phages from STEC O26:H11

**DOI:** 10.3389/fmicb.2017.00296

**Published:** 2017-03-03

**Authors:** Ludivine Bonanno, Benjamin Delubac, Valérie Michel, Frédéric Auvray

**Affiliations:** ^1^Université Paris-Est, Anses, Laboratory for Food SafetyMaisons-Alfort, France; ^2^ACTALIA Produits Laitiers, Laboratoire de Microbiologie d’Intérêt LaitierLa Roche sur Foron, France

**Keywords:** Stx phages, STEC, O26:H11, cheese, induction

## Abstract

Shiga toxin-producing *Escherichia coli* (STEC) are responsible for human infections, ranging from mild watery diarrhea to hemorrhagic colitis (CH) that may be complicated by hemolytic uremic syndrome (HUS). The main STEC virulence factor is Shiga toxin encoded by the *stx* gene, located in the genome of a bacteriophage integrated into the bacterial chromosome. The serotype O26:H11 is the second HUS-causing serotype worldwide (after O157:H7), and the first found in dairy products such as raw-milk cheeses. A small number of HUS cases identified each year in France are caused by serotype O26:H11. Stx phage induction is known to result in STEC lysis and release of new Stx phages particles. This phenomenon could negatively impact STEC screening in foods based on *stx* gene detection by PCR. Here, we evaluated the influence of physicochemical parameters related to cheese-making process on the induction rate of Stx phages from STEC O26:H11, including H_2_O_2_, NaCl, lactic acid and temperature. In addition, selective agents from the analytical STEC enrichment and detection procedure (XP CEN ISO/TS 13136) were tested, including novobiocin, acrifavin, cefixim-tellurite, and bile salts. An impact of H_2_O_2_ and NaCl on Stx phage induction was observed. Production of Stx phages was also observed during a real cheese-making process. By contrast, no significant effect could be demonstrated for the chemical agents of the STEC detection procedure when tested separately, except for acriflavin and novobiocin which reduced Stx1 phage production in some cases. In conclusion, these results suggest that the cheese-making process might trigger the production of Stx phages, potentially interfering with the analysis of STEC in food.

## Introduction

Shiga toxin-producing *Escherichia coli* (STEC) O26:H11 were first identified as causes of hemolytic uremic syndrome (HUS) in 1983 (Karmali et al., 1983; Tarr et al., 2005). They correspond to one of the most commonly isolated non-O157:H7 serotype worldwide, accounting for 12% of all clinical enterohemorrhagic *E. coli* (EHEC) in Europe in 2012 (Zimmerhackl et al., 2010; EFSA, 2014) and for 22% of clinical non-O157 EHEC isolates in the United States between 1983 and 2002 (Brooks et al., 2005). Transmission of STEC to humans occurs through food, water and direct contact with animals and their environment. In 2005, in France, STEC O26:H11 was involved in an outbreak that included 16 HUS cases and was linked to consumption of contaminated unpasteurized Camembert cheese (Espie et al., 2006). Since the early 2000s, the French institute for public health surveillance has observed a significant increase in France in the proportion of reported HUS cases due to non-O157 serogroups, with 16% of HUS cases caused by the serogroup O26 over the period 1996–2014 (InVS, 2014).

Shiga toxin, the main virulence factor of STEC, is encoded by *stx* genes within the genome of a prophage (Stx phage) located in the bacterial chromosome (Smith et al., 1983; O’Brien et al., 1984; Schmidt, 2001). Two Stx groups, Stx1 and Stx2, have been identified (Scheutz et al., 2012). The first Stx1 phage described was phage H19B which was isolated from a clinical EHEC O26 strain (Smith et al., 1983). Stx phages are inducible from the host strain by DNA-damaging agents such as antibiotics (Kimmitt et al., 2000; Kohler et al., 2000), which trigger the SOS response of *E. coli* (Little and Mount, 1982) and result in the derepression of phage lytic genes, production of phage particles, lysis of the bacterial host cells and release of the phage particles. Recently we described the induction of Stx phages from STEC O26:H11 by mitomycin C, and showed that Stx2 phages were more inducible than Stx1 phages (Bonanno et al., 2016).

Exposure of STEC to other stressful agents such as NaCl, temperature, hydrogen peroxide and pH can also lead to Stx prophage activation (Los et al., 2009, 2010; Harris et al., 2012; Imamovic and Muniesa, 2012). Stx phage induction in food could result in the presence of free phage particles (Imamovic and Muniesa, 2011). Consequently, the presence of free Stx phage particles could lead to the production of false presumptive STEC-positive results when food samples are identified as “*stx*-positive” by PCR. In addition, these Stx phages could infect other *E. coli* strains and convert them into pathogenic bacteria. Moreover, excision of Stx prophages might result in the isolation of *stx-*negative *E. coli* originating from STEC.

In Europe, official controls of STEC in food samples are carried out according to the technical specification XP CEN ISO/TS 13136 (ISO, 2012). This method includes an enrichment step in the presence of selective agents (such as bile salts, novobiocin, or acriflavin) favoring STEC development over background microorganisms. Imamovic and Muniesa (2011) demonstrated a significant increase in the densities of Stx phages after enrichment for 52–56% of minced beef samples and 39–65% of salad samples (Imamovic and Muniesa, 2011). Again, the presence of these free Stx phages might interfere with the analysis of food samples for contamination by STEC. Testing food samples for the presence of STEC using PCR targeting the *stx* gene can lead to a high amount of *stx*-positive samples (*ca* 30%) which are not all subsequently confirmed by the isolation of STEC colony (Fach et al., 2001; Vernozy-Rozand et al., 2005; Madic et al., 2011; Trevisani et al., 2014). In addition, *stx*-negative *E. coli* also named attaching and effacing *E. coli* (AEEC) are also frequently isolated from foods (Anses, 2012; Trevisani et al., 2014). These could derive from STEC by loss of Stx phage during the isolation step since STEC O26:H11 strains were demonstrated to frequently lose and acquire Stx phages (Karch et al., 1992; Bielaszewska et al., 2007). Official surveys performed in France in 2009 (Anses, 2012) highlighted the isolation of an equivalent proportion of STEC and AEEC strains in 1911 raw milk cheeses samples, i.e., 15 and 17 strains, respectively (Anses, 2012). Madic et al. (2011) also showed STEC and AEEC O26:H11 could be isolated from *stx*-positive samples of raw-milk cheese (400 samples analyzed), i.e., seven and three strains, respectively. Finally, *stx*-positive and *stx*-negative *E. coli* O26 were isolated from milk (i.e., 0.4 and 2% samples, respectively) and milk filters (i.e., 0.4 and 2% filters, respectively) in Italy (Trevisani et al., 2014).

This study aimed at investigating whether Stx phage induction and release could occur from STEC O26:H11 in two different situations, i.e., (i) during cheese manufacturing and (ii) during the use of STEC detection procedure. The level of Stx phage induction, from three STEC O26:H11 strains, was analyzed in experimental conditions related to the cheese-making process and to the analytical STEC detection procedure. Induction levels of Stx1 and Stx2 phages were quantified by qPCR and compared to each other.

## Materials and Methods

### Bacterial Strains

Three STEC O26:H11 strains (2976-1, F46-223, and 09QMA-277.2) (Bonanno et al., 2016), isolated from dairy products and containing *stx1*, *stx2* and both *stx1* and *stx2* genes, respectively, were used in this study. *E. coli* strains were cultivated in Lysogeny broth (LB) at 37°C.

### Bacteriophage Induction

An overnight culture of STEC O26:H11 was inoculated at 2% in a fresh LB medium with 5 mM of CaCl_2_ and incubated at 37°C. At the exponential growth phase (OD_600_ of 0.3), cultures were further incubated at 37°C for 24 h with shaking at 240 rpm, in the presence of stress factors (listed below). All cultures were centrifuged at 7,200 × *g* for 10 min, and the supernatants were filtered through low-protein-binding 0.22 μm-pore-size membrane filters (Millex-GP PES; Millipore, St-Quentin-en-Yvelines, France) for phage purification.

The factors linked to cheese manufacturing that were studied for their impact on Stx phage induction were lactic acid at 0.05, 0.5, 1.5, and 3%, hydrogen peroxide (H_2_O_2_) at 0.25 and 3 mM, salt (NaCl) at a final concentration of 3% (taking into account NaCl from the LB broth). The effect of temperature, i.e., 9 and 42°C, was also tested.

The other studied factors were related to the analytical STEC detection procedure and included acriflavin (12 mg/l), novobiocin (20 mg/l) and bile salts (1.5 g/l). Finally, cefixime-tellurite (C-T; 0.05 and 2.5 mg/l), used as a supplement in the Rhamnose MacConkey agar (RMAC) adapted for the isolation of STEC O26 (Hiramatsu et al., 2002) was also evaluated for its ability to induce Stx phage.

All these experiments were performed in duplicate. Moreover, each strain was also cultured without inducing agent at 37°C as a control representing the spontaneous induction of Stx phages. Twelve to 14 replicates (*n*_R_) per strain were performed. Bacteriophage spontaneous induction variability, expressed in log_10_ *stx* gene copies per milliliter (GCs/ml), was characterized for each strain with normal distribution adjusted to *n*_R_ values. The effect of a factor was assessed by calculating the probability of observing log_10_ (GCs/ml) if the factor has no effect on induction (Prob_i/s_), i.e., if the production of Stx phage is only due to spontaneous induction. A factor was considered to significantly induce more Stx phages compared to spontaneous induction when both Prob_i/s_ values (associated to replicate values) were above 0.975. A factor was considered to significantly induce less Stx phages compared to spontaneous induction when both Prob_i/s_ values were below 0.025.

### Quantification of Stx Phage

Filtered supernatants obtained after Stx phage induction were treated with DNase using the Turbo DNA-free^TM^ kit (Ambion^®^, life technologies, Illkirch, France) and phage DNA was released by heat treatment for 10 min at 100°C (Bonanno et al., 2016). Quantitative PCR (qPCR) assays targeting *stx1* and *stx2* genes, were then used with the LightCycler^®^ 480 instrument (Roche Diagnostics, Meylan, France) as described previously (Derzelle et al., 2011). However, the fluorescent reporter Flc of the *stx1* probe was replaced by Rox to eliminate any interference arising from the acriflavin solution.

DNA from strain EDL933 which carries only one copy of the *stx1* and *stx2* genes was used for preparation of the standard curve. Briefly, bacterial cells were collected by low speed centrifugation, and supernatants containing any spontaneously induced Stx phages were discarded. Genome DNA was then purified from the pelleted bacterial cells and its concentration determined using a Nanodrop spectrophotometer (Thermo Scientific, Illkirch, France). Genomic copy numbers were calculated from the known size of EDL933 genome (i.e., 5.44 Mb). The standard curve was prepared from serially diluted genomic DNA and used for the quantification of Stx phages expressed as log_10_ *stx* gene copies per milliliter (GCs/ml). All the samples were run together with the standard-positive and negative controls. Moreover, the absence of inhibition was verified by the absence of Ct variation of the standard in the presence of DNA extracts prepared from the studied stress conditions.

## Results and Discussion

### Impact of Cheese-Making Process on Stx Phage Induction

Four factors related to the manufacturing of cheeses were selected in this study and tested for their ability to induce Stx phages. Lactic acid (at various concentrations) was used to mimic acid stress provoked by lactic acid bacteria during the coagulation step. Salt (NaCl) at 3% was tested due to its role in the conservation and flavor enrichment step. Hydrogen peroxide (H_2_O_2_, at various concentrations) was used to mimic oxidative stress caused by other bacteria present in the cheese matrix. Finally, the initial milk heating step and final ripening (cooling) step were tested by performing bacterial growth at 42 and 9°C, respectively.

**FIGURE 1:**
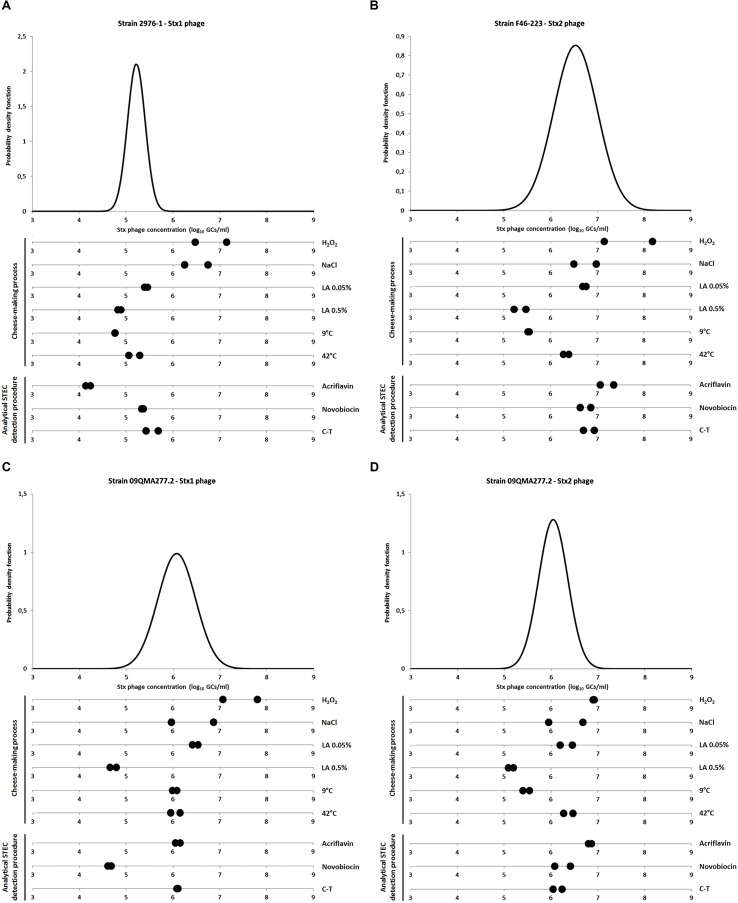
**Quantification of Stx phages after exposure to stress factors.** **(A)** Stx1 phage production from strain 2976-1; **(B)** Stx2 phage production from strain F46-223; **(C,D)** Stx1 and Stx2 phages production, respectively, from strain 09QMA277.2. Stx phages were quantified by qPCR and expressed as log_10_ stx gene copies per milliliter (GCs/ml). The variability of Stx phage spontaneous induction was characterized for each strain with normal distribution adjusted to n_R_ values (corresponding to 12–14 replicates per strain). Stress factors tested were either related to cheese making process [H_2_O_2_ 3 mM, 3% NaCl, Lactic acid (LA) 0.05 and 0.5%, 9 and 42°C] or related to the analytical STEC detection procedure [acriflavin 12 mg/l, novobiocin 20 mg/l and cefixime-tellurite (C-T) at 0.05 and 2.5 mg/l, respectively]. All these experiments were performed in duplicate.

**Figure 1** presents the amount of Stx phages produced by each strain in the presence of the factors tested compared to the control representing the spontaneous induction of Stx phages (i.e., without inducing agent).

When STEC were grown in the presence of H_2_O_2_ to 3 mM, an increase in the concentrations of Stx phages was significantly observed for strains 2976-1 (**Figure 1A**) and 09QMA277.2 (**Figures 1C,D**). For strain F46-223, only one of the two replicate showed a significant induction effect (**Figure 1B**). Therefore, we considered that H_2_O_2_ at 3 mM induced Stx phages *in vitro*. This result was in agreement with previous reports which described the inducing effect on Stx phages by H_2_O_2_ 3 mM (Los et al., 2009, 2010).

Interestingly, 3% NaCl induced Stx1 phage only from strain 2976-1 (**Figure 1A**), but this was not the case for the Stx2 phage neither from strain F46-223, nor for both Stx1 and Stx2 phages from strain 09QMA277.2 (**Figures 1B–D**). Harris et al. (2012) described that 3% NaCl inhibited Stx phage induction presumably because of the inhibitory effect of 3% salt on vital physiological processes. On the other hand, they also demonstrated that the presence of a salt concentration (2%) equivalent to that found for meat processing induced Stx phages (Harris et al., 2012). Moreover, Wagner et al. (2002) demonstrated that Stx1 phages induction could be regulated by RecA-independent pathway and therefore not by the SOS response. In our study, Stx phage induction did not occur in the presence of 3% NaCl except for an Stx1 phage from one strain. Whether the alternative RecA-independent induction system was used in this strain remains to be determined. This phenomenon was not observed for the Stx1 phage from strain 09QMA277.2 which possesses two Stx phages (Stx1 and Stx2). However, it was shown previously that when two Stx prophages are integrated into the bacterial chromosome, the production of Shiga-toxin and the activation rate of the lytic cycle of each phage are both highly reduced (Muniesa et al., 2003; Serra-Moreno et al., 2008).

The presence of lactic acid at 0.05% resulted in Stx phage production similar to the spontaneous induction (**Figure 1**) and a slight decrease in Stx phage production was even observed with 0.5% lactic acid (**Figure 1**). This is in agreement with a previous study which showed that for pH lower than 5.5, inhibition of the induction of Stx phages occurred, even in the presence of mitomycin C (Imamovic and Muniesa, 2012). In addition, *stx* gene transcription was shown to be very low or non-existent at pH 5.5 (Olesen and Jespersen, 2010), consistent with Stx phage repression at low pH. This phenomenon could be linked to the RpoS system involved in the survival of bacteria triggered by acid stress (Cheville et al., 1996). No Stx phages could be detected when higher concentrations of lactic acid such as 1.5 and 3% were used but in both cases this was due to the inhibition of PCR.

Incubation of STEC at a low temperature (9°C) or at 42°C did not reveal significantly difference with the spontaneous induction (**Figure 1**) A previous study showed that the temperature had an effect on the induction of Stx phage, which was lower at 30°C than at 37°C whereas at 43°C, it was considerably increased (Los et al., 2009). However, this observation was made in the presence of inducing agents such as mitomycin C, UV irradiation and H_2_O_2_, i.e., in experimental conditions that differ from those tested here.

Finally, Stx phage induction was also tested in real cheese-making conditions where cheeses were produced in an experimental plant using milk inoculated with an *stx1*- or *stx2*-positive STEC O26:H11 strain. A total of 48 samples were collected at various time points (i.e., 6 h, 24 h, 8 and 28 days) during cheese production. Stx1 and Stx2 phages were detected from 3 and 7 samples, respectively. No correlation between the induction of Stx phage and the different sampling steps during the process could be observed.

### Impact of STEC Detection Procedure on Stx Phage Induction

The analytical STEC detection procedure based on the technical specification XP CEN ISO/TS 13136 relies on the use of several selective agents. Acriflavin (at 12 mg/l) and novobiocin (at 20 mg/l) are used for STEC isolation from dairy products and other food categories, respectively. They are added to the enrichment medium, triptic soy broth (TSB) modified with bile salts at 1.5 g/l (mTSB). Moreover, the supplement cefixime-tellurite (C-T, at 0.05 and 2.5 mg/l, respectively) is used as a selective agent in agar medium for the isolation of STEC O26 onto Rhamnose MacConkey agar (CT-RMAC) (Hiramatsu et al., 2002). These specific culture media allow the enrichment and isolation of STEC to the detriment of the background microflora.

The presence of C-T had no effect on Stx phage induction (**Figure 1**). It was also the case for novobiocin and acriflavin (**Figure 1**), with two exceptions. First, Stx1 phage production from 09QMA277.2 was considerably reduced in the presence of novobiocin compared to the control condition (**Figure 1C**). Then, Stx1 phage production from 2976-1 strain was also reduced but this occurred in the presence of acriflavin (**Figure 1A**). An additional test was performed therefore with two other *stx1-*positive STEC O26:H11 strains (i.e., 10d and 09QMA245.2). A decrease of Stx1 phage production was also observed in the presence of acriflavin but only for one of the two strains (i.e., 10d) (data not shown). Distinct genetic backgrounds of the various strains tested could explain the differences observed in Stx phage production in response to acriflavin. This selective agent was shown to induce cell wall changes in *Staphylococcus aureus* (Kawai and Yamagishi, 2009). Moreover, it has the capacity to bind on the cell wall of *E. coli* and the acriflavin-binding capacity is controlled by the *acrA* gene. A mutation of *acrA* leads to sensitivity not only to acriflavin but also to mitomycin C (Nakamura and Shinya, 1985). This observation may therefore suggest differences in the *acrA* gene and acriflavin sensitivity for 2976-1 and 10d strains compared to 09QMA277.2 and 09QMA245.2 strains. Moreover, such differences would also explain the higher production of Stx1 phage observed elsewhere in the presence of mitomycin C for strains 2976-1 and 10d compared to strains 09QMA277.2 and 09QMA245.2 (Bonanno et al., 2016).

Finally, induction of Stx phage by bile salts at 1.5 g/l could not be quantified by qPCR due to the inhibition of PCR by bile salts. However, a slight decrease of OD_600_ (of -0.13 to -0.57 units) was observed with bile salts at 1.5 g/l for the three strains tested (data not shown) which might reflect induction of Stx phages.

## Conclusion

In this study, we demonstrated that oxidative stress and, to a lesser extent, salt stress, both occurring during cheese-making processes, have the ability to induce Stx phages *in vitro*. Moreover, production of Stx phages was also observed during a real cheese-making process when milk was inoculated by a STEC O26:H11 strain. These observations suggest that Stx phages could be present as free particles in cheeses and could infect other *E. coli* or enterobacterial species from the microflora in the cheese matrix or inside the human gut after consumption. These free Stx phages could also contribute to the production of *stx*-positive signals obtained during PCR-based screening of STEC in foods, explaining the reported difficulties to isolate STEC from *stx*-positive food samples.

Concerning the analytical STEC detection procedure based on the technical specification XP CEN ISO/TS 13136, no significant effect on Stx phage induction was observed. Consequently, this lack of induction suggests that AEEC isolated from *stx*-positive food samples are unlikely to derive from STEC by loss of their Stx phage during the enrichment or isolation procedure. AEEC might therefore simply co-exist with STEC in food and then overgrow STEC, leading to their isolation to the detriment of STEC. However, it should be noted that the chemical and temperature factors were all tested here separately, and these could act synergistically to induce Stx phages, as previously shown for mitomycin C and EDTA tested together (Imamovic and Muniesa, 2012). Combining these factors is therefore needed before to conclude definitely on the impact of the enrichment and isolation steps on Stx phage induction. Whether STEC lysis is also triggered upon Stx phage induction during the whole analytical procedure (i.e., when all the factors are combined) and thus contribute to STEC isolation failure remains to be further investigated.

## Author Contributions

LB contributed to conception, design, data acquisition, analysis, and interpretation, drafted and critically revised the manuscript. BD contributed to design, data acquisition, analysis and interpretation. VM contributed to conception, data interpretation, and critically revised the manuscript. FA contributed to conception, data interpretation, drafted and critically revised the manuscript.

## Conflict of Interest Statement

The authors declare that the research was conducted in the absence of any commercial or financial relationships that could be construed as a potential conflict of interest.

The reviewer MLDG and handling Editor declared their shared affiliation and the handling Editor states that the process nevertheless met the standards of a fair and objective review.

## References

[B1] Anses (2012). Surveillance des *E. coli* producteurs de shigatoxines (STEC) dans les denrées alimentaires en France (2005-2011). *Bull. Épidémiol.* 55 3–9.

[B2] Bielaszewska M. Prager R. Kock R. Mellmann A. Zhang W. Tschape H. (2007). Shiga toxin gene loss and transfer in vitro and in vivo during enterohemorrhagic *Escherichia coli* O26 infection in humans. *Appl. Environ. Microbiol.* 73 3144–3150. 10.1128/AEM.02937-06 17400784 PMC1907125

[B3] Bonanno L. Petit M. A. Loukiadis E. Michel V. Auvray F. (2016). Heterogeneity in induction level, infection ability, and morphology of shiga toxin-encoding phages (Stx phages) from dairy and human shiga toxin-producing *Escherichia coli* O26:H11 isolates. *Appl. Environ. Microbiol.* 82 2177–2186. 10.1128/AEM.03463-15 26826235 PMC4807521

[B4] Brooks J. T. Sowers E. G. Wells J. G. Greene K. D. Griffin P. M. Hoekstra R. M. (2005). Non-O157 Shiga toxin-producing *Escherichia coli* infections in the United States, 1983-2002. *J. Infect. Dis.* 192 1422–1429. 10.1086/466536 16170761

[B5] Cheville A. M. Arnold K. W. Buchrieser C. Cheng C. M. Kaspar C. W. (1996). rpoS regulation of acid, heat, and salt tolerance in *Escherichia coli* O157:H7. *Appl. Environ. Microbiol.* 62 1822–1824. 8633882 10.1128/aem.62.5.1822-1824.1996 PMC167958

[B6] Derzelle S. Grine A. Madic J. de Garam C. P. Vingadassalon N. Dilasser F. (2011). A quantitative PCR assay for the detection and quantification of Shiga toxin-producing *Escherichia coli* (STEC) in minced beef and dairy products. *Int. J. Food Microbiol.* 151 44–51. 10.1016/j.ijfoodmicro.2011.07.039 21878400

[B7] EFSA (2014). The European Union summary report on trends and sources of zoonoses, zoonotic agents and food-borne outbreaks in 2012. *EFSA J.* 12:3547 10.2903/j.efsa.2014.3547 PMC7009540 32625785

[B8] Espie E. Mariani-Kurkdjian P. Grimont F. Pithier N. Vaillant V. Francart S. (2006). “Shiga-toxin producing *Escherichia coli* O26 infection and unpasteurised cows cheese, France 2005” in *Proceedings of the 6th International Symposium on Shiga Toxin (Verocytoxin) – Producing Escherichia coli infections*, ed. Sofronidis J. (Melbourne, VIC). Available at: http://opac.invs.sante.fr/doc_num.php?explnum_id=7011

[B9] Fach P. Perelle S. Dilasser F. Grout J. (2001). Comparison between a PCR-ELISA test and the vero cell assay for detecting Shiga toxin-producing *Escherichia coli* in dairy products and characterization of virulence traits of the isolated strains. *J. Appl. Microbiol.* 90 809–818. 10.1046/j.1365-2672.2001.01313.x 11348443

[B10] Harris S. M. Yue W. F. Olsen S. A. Hu J. Means W. J. McCormick R. J. (2012). Salt at concentrations relevant to meat processing enhances Shiga toxin 2 production in *Escherichia coli* O157:H7. *Int. J. Food Microbiol.* 159 186–192. 10.1016/j.ijfoodmicro.2012.09.007 23107496

[B11] Hiramatsu R. Matsumoto M. Miwa Y. Suzuki Y. Saito M. Miyazaki Y. (2002). Characterization of Shiga toxin-producing *Escherichia coli* O26 strains and establishment of selective isolation media for these strains. *J. Clin. Microbiol.* 40 922–925. 10.1128/JCM.40.3.922-925.2002 11880417 PMC120279

[B12] Imamovic L. Muniesa M. (2011). Quantification and evaluation of infectivity of shiga toxin-encoding bacteriophages in beef and salad. *Appl. Environ. Microbiol.* 77 3536–3540. 10.1128/AEM.02703-10 21441341 PMC3126450

[B13] Imamovic L. Muniesa M. (2012). Characterizing RecA-independent induction of Shiga toxin2-encoding phages by EDTA treatment. *PLoS ONE* 7:e32393 10.1371/journal.pone.0032393 PMC3290563 22393404

[B14] InVS (2014). *Surveillance du Syndrome Hémolytique et Urémique Post-Diarrhéique Chez les Enfants de Moins de 15 ans en France en 2014*. Paris: Institut Pasteur.

[B15] ISO (2012). *ISO/TS 13136:2012 Microbiologie des Aliments – Méthode Basée sur la Réaction de Polymérisation en Chaîne (PCR) en Temps Réel Pour la Détection des Micro-Organismes Pathogènes dans les Aliments – Méthode Horizontale Pour la Détection des Escherichia coli Producteurs de Shigatoxines (STEC) et la Détermination des Sérogroupes O157 O111 O26 O103 et O145*. Available at: http://www.iso.org/iso/fr/iso_catalogue/catalogue_tc/catalogue_detail.htm?csnumber=53328

[B16] Karch H. Meyer T. Russmann H. Heesemann J. (1992). Frequent loss of Shiga-like toxin genes in clinical isolates of *Escherichia coli* upon subcultivation. *Infect. Immun.* 60 3464–3467. 1639518 10.1128/iai.60.8.3464-3467.1992 PMC257340

[B17] Karmali M. A. Steele B. T. Petric M. Lim C. (1983). Sporadic cases of haemolytic-uraemic syndrome associated with faecal cytotoxin and cytotoxin-producing *Escherichia coli* in stools. *Lancet* 1 619–620. 10.1016/S0140-6736(83)91795-6 6131302

[B18] Kawai M. Yamagishi J. (2009). Mechanisms of action of acriflavine: electron microscopic study of cell wall changes induced in *Staphylococcus aureus* by acriflavine. *Microbiol. Immunol.* 53 481–486. 10.1111/j.1348-0421.2009.00151.x 19703241

[B19] Kimmitt P. T. Harwood C. R. Barer M. R. (2000). Toxin gene expression by shiga toxin-producing *Escherichia coli*: the role of antibiotics and the bacterial SOS response. *Emerg. Infect. Dis.* 6 458–465. 10.3201/eid0605.000503 10998375 PMC2627954

[B20] Kohler B. Karch H. Schmidt H. (2000). Antibacterials that are used as growth promoters in animal husbandry can affect the release of Shiga-toxin-2-converting bacteriophages and Shiga toxin 2 from *Escherichia coli* strains. *Microbiology* 146(Pt 5), 1085–1090. 10.1099/00221287-146-5-1085 10832635

[B21] Little J. W. Mount D. W. (1982). The SOS regulatory system of *Escherichia coli*. *Cell* 29 11–22. 10.1016/0092-8674(82)90085-X 7049397

[B22] Los J. M. Los M. Wegrzyn A. Wegrzyn G. (2010). Hydrogen peroxide-mediated induction of the Shiga toxin-converting lambdoid prophage ST2-8624 in *Escherichia coli* O157:H7. *FEMS Immunol. Med. Microbiol.* 58 322–329. 10.1111/j.1574-695X.2009.00644.x 20070366

[B23] Los J. M. Los M. Wegrzyn G. Wegrzyn A. (2009). Differential efficiency of induction of various lambdoid prophages responsible for production of Shiga toxins in response to different induction agents. *Microb. Pathog.* 47 289–298. 10.1016/j.micpath.2009.09.006 19761828

[B24] Madic J. Vingadassalon N. de Garam C. P. Marault M. Scheutz F. Brugere H. (2011). Detection of Shiga toxin-producing *Escherichia coli* serotypes O26:H11, O103:H2, O111:H8, O145:H28, and O157:H7 in raw-milk cheeses by using multiplex real-time PCR. *Appl. Environ. Microbiol.* 77 2035–2041. 10.1128/AEM.02089-10 21239543 PMC3067316

[B25] Muniesa M. de Simon M. Prats G. Ferrer D. Panella H. Jofre J. (2003). Shiga toxin 2-converting bacteriophages associated with clonal variability in *Escherichia coli* O157:H7 strains of human origin isolated from a single outbreak. *Infect. Immun.* 71 4554–4562. 10.1128/IAI.71.8.4554-4562.2003 12874335 PMC166033

[B26] Nakamura H. Shinya T. (1985). Acriflavine-binding capacity controlled by the acrA gene of *Escherichia coli*. *J. Gen. Microbiol.* 131 1639–1647. 10.1099/00221287-131-7-1639 3900282

[B27] O’Brien A. D. Newland J. W. Miller S. F. Holmes R. K. Smith H. W. Formal S. B. (1984). Shiga-like toxin-converting phages from *Escherichia coli* strains that cause hemorrhagic colitis or infantile diarrhea. *Science* 226 694–696. 10.1126/science.6387911 6387911

[B28] Olesen I. Jespersen L. (2010). Relative gene transcription and pathogenicity of enterohemorrhagic *Escherichia coli* after long-term adaptation to acid and salt stress. *Int. J. Food Microbiol.* 141 248–253. 10.1016/j.ijfoodmicro.2010.05.019 20603024

[B29] Scheutz F. Teel L. D. Beutin L. Pierard D. Buvens G. Karch H. (2012). Multicenter evaluation of a sequence-based protocol for subtyping Shiga toxins and standardizing Stx nomenclature. *J. Clin. Microbiol.* 50 2951–2963. 10.1128/JCM.00860-12 22760050 PMC3421821

[B30] Schmidt H. (2001). Shiga-toxin-converting bacteriophages. *Res. Microbiol.* 152 687–695. 10.1016/S0923-2508(01)01249-9 11686382

[B31] Serra-Moreno R. Jofre J. Muniesa M. (2008). The CI repressors of Shiga toxin-converting prophages are involved in coinfection of *Escherichia coli* strains, which causes a down regulation in the production of Shiga toxin 2. *J. Bacteriol.* 190 4722–4735. 10.1128/JB.00069-08 18469095 PMC2446792

[B32] Smith H. W. Green P. Parsell Z. (1983). Vero cell toxins in *Escherichia coli* and related bacteria: transfer by phage and conjugation and toxic action in laboratory animals, chickens and pigs. *J. Gen. Microbiol.* 129 3121–3137. 10.1099/00221287-129-10-3121 6418852

[B33] Tarr P. I. Gordon C. A. Chandler W. L. (2005). Shiga-toxin-producing *Escherichia coli* and haemolytic uraemic syndrome. *Lancet* 365 1073–1086. 10.1016/S0140-6736(05)71144-2 15781103

[B34] Trevisani M. Mancusi R. Delle Donne G. Bacci C. Bassi L. Bonardi S. (2014). Detection of Shiga toxin (Stx)-producing *Escherichia coli* (STEC) in bovine dairy herds in Northern Italy. *Int. J. Food Microbiol.* 184 45–49. 10.1016/j.ijfoodmicro.2013.12.033 24495690

[B35] Vernozy-Rozand C. Montet M. P. Berardin M. Bavai C. Beutin L. (2005). Isolation and characterization of Shiga toxin-producing *Escherichia coli* strains from raw milk cheeses in France. *Lett. Appl. Microbiol.* 41 235–241. 10.1111/j.1472-765X.2005.01756.x 16108913

[B36] Wagner P. L. Livny J. Neely M. N. Acheson D. W. Friedman D. I. Waldor M. K. (2002). Bacteriophage control of Shiga toxin 1 production and release by *Escherichia coli*. *Mol. Microbiol.* 44 957–970. 10.1046/j.1365-2958.2002.02950.x 12010491

[B37] Zimmerhackl L. B. Rosales A. Hofer J. Riedl M. Jungraithmayr T. Mellmann A. (2010). Enterohemorrhagic *Escherichia coli* O26:H11-associated hemolytic uremic syndrome: bacteriology and clinical presentation. *Semin. Thromb. Hemost.* 36 586–593. 10.1055/s-0030-1262880 20865635

